# Opening up with COVID-19 passes

**DOI:** 10.2471/BLT.21.020821

**Published:** 2021-08-01

## Abstract

Governments worldwide are pressing ahead with COVID-19 passes, despite significant technical, ethical and social obstacles to implementation. Gary Humphreys reports.

Passengers at the Roadport bus terminal in central Harare, Zimbabwe are used to showing their vaccination certification cards before they get on the bus.

“The cards were introduced in the late 1960s as a measure to control the spread of yellow fever,” explains Dr Integrity Mchechesi, Harare-based co-founder of Vaxiglobal, a Zimbabwean health-tech start-up focused on digital information solutions for health systems. The cards, containing the International Certificate of Vaccination or Prophylaxis, issued by the World Health Organization (WHO), are required for Zimbabweans crossing into neighbouring countries such as Mozambique or the United Republic of Tanzania whether for business or personal reasons.

What Zimbabweans (and people in other countries with endemic yellow fever) have been doing for more than half a century, people worldwide are now starting to embrace for internal purposes as well as for international travel, presenting passes that are intended to indicate the reduced likelihood of their catching or spreading coronavirus disease 2019 (COVID-19).

“There has been enormous pressure to introduce COVID-19 passports or passes to support the opening up of countries after protracted social and economic lockdowns,” says Imogen Parker, associate director of policy at the Ada Lovelace Institute, a nongovernmental organization that is monitoring the development and roll-out of vaccination passes and COVID-19 status apps.

That pressure has resulted in multiple country-level initiatives to introduce such documents for internal purposes as well as international travel. For example, the Green Pass system introduced by Israel in February gives holders access to theatres, concert halls, restaurants and bars as well as unrestricted travel to Greece. Other early adopters include New York State in the United States of America, which introduced its Excelsior Pass system in March, and Denmark which introduced its Coronapas system in April.

According to Parker, more is to come. “Indications of gathering momentum include regional initiatives such as that being implemented in the European Union; digital certificates to indicate whether a person has been vaccinated against COVID-19, received a negative test result or recovered from the virus,” she says.

The African Union Commission and the Africa Centres for Disease Control and Prevention (Africa CDC) are developing a ‘My COVID Pass’ tool to simplify verification of public health documentation for travellers crossing national borders. The tool is part of a Trusted Travel initiative which will include an Africa CDC mutual recognition protocol for COVID-19 testing and test results, and vaccination certificates (including certificates for yellow fever and COVID-19).

“The long-term effects of building such systems […] must be considered.”

Imogen Parker

The speed with which pass systems are being rolled out makes some people uneasy. Parker is one of them. “Everyone is understandably keen to open up, and COVID-19 passes seem like an obvious way to do that, but there are good reasons to pause for reflection,” she says.

Others question whether the passes are in fact fit for purpose. “The evidence on the level and duration of protection of people who have recovered from COVID-19, from reinfection or infection with a new variant is still evolving, which puts in question the validity of immunity passes and the ethical basis for their use,” says Dr Andreas Reis, Co-Lead of the Health Ethics & Governance Unit at the World Health Organization (WHO).

Reis has similar concerns about passes based on vaccination status. “The extent to which each vaccine prevents transmission of different SARS-CoV-2 (severe acute respiratory syndrome coronavirus 2) variants remains to be assessed and it is not known how long each vaccine confers protection on individuals or the extent to which vaccine effectiveness might be affected by new variants,” he says.

Though concerned about these issues, Reis is quick to point out that the public health challenges posed by COVID-19 passes go beyond the question of their efficacy. “The potential for discriminatory treatment of unvaccinated individuals is also of concern, as is the unequitable access to digital certificates in countries where a significant portion of the population lacks access to information and communications technology.”

At a more nuts-and-bolts level, achieving interoperability between disparate digital systems also presents a challenge. Despite regional initiatives of the kind mentioned above, the emerging COVID-19 pass landscape is generally characterized by multiple ad hoc initiatives, many of them driven by private sector companies such as airlines. These interoperability problems are only exacerbated in pass systems intended for use by international travellers.

“One of the major global problems we face is that common standards and governance for security, authentication, privacy and health data exchange have yet to be agreed on,” says Bernardo Mariano, Chief Information Technology Officer at the United Nations.

To help address the problem, WHO has brought together a consortium of experts to focus on defining specifications and standards for digital vaccination certification and laboratory analyses related to SARS-CoV-2 test results and Covid-19 recovery status (serological status) that will serve current and future requirements of digital systems. A core consortium aim is to support cross-border implementation. According to Mariano, the consortium will be publishing guidance in the coming months.

The security of digital pass systems is also under scrutiny. Parker is concerned that systems carrying important health data are not only susceptible to hacking, but risk being compromised by commercial stakeholders seeking to expand digital and identity infrastructure.

For Parker, such security questions feed into deeper societal concerns about equity and liberty. For this reason, she advocates policy-driven development informed by fully inclusive national dialogue. “It’s really important that this doesn’t just happen organically, with a number of private sector stakeholders introducing pass systems to suit their own requirements,” she cautions.

Parker is also worried about what she calls “scope creep”, the potential extension of the pass remit to cover other health risk factors such as tobacco use, HIV status or mental health status, or their use for non-health purposes such as policing. “The long-term effects of building such systems and how they could shape the future must be considered,” she says.

“Common standards and governance […] have yet to be agreed on.”

Bernardo Mariano

But what about the short term? What can we do now, in the face of what is a global emergency? Parker advocates comparing the risks and benefits of introducing novel technologies with the risks and benefits of the public health tools that are already available.

“For countries that are approaching herd immunity, the hope is that COVID-19 becomes comparable in impact to seasonal influenza. While this is clearly not influenza, we nevertheless have to ask ourselves why our approach to tackling COVID-19 should be so radically different,” she says.

Clearly, this is an argument that carries more weight in countries with relatively robust vaccination campaigns, where living conditions allow for social distancing, and where teleworking and government support have thus far allowed economies to struggle along, notwithstanding the devastation of certain sectors.

In countries where these conditions do not apply, the argument becomes harder to make – countries like Zimbabwe, where just under 4% of the total population (555 277 of 14.5 million) has been fully vaccinated, living conditions in informal settlements in and around the capital make social distancing difficult, and the majority of the population has no access to a social safety net or teleworking. For many of the people in such countries, physically going to work is a vital necessity and for some that means getting on a bus.

Can passes make that activity safer? Perhaps, but the implementation challenges are significant, starting with ensuring that the passes used are not fakes.

“Paper-based systems are subject to falsification, as shown by the use of the yellow cards in Zimbabwe,” says Mchechesi, who went to the Roadport terminal in May of 2019 with a team of researchers to see how many of the international vaccination certification cards were fake. “We were expecting a ratio of around 50% but found that roughly 80% of the cards were falsified,” he says, adding that since that time, a market in fake COVID-19 test certificates has also flourished at the terminal.

Mchechesi is hoping to help fix the problem with Vaxiglobal’s digital verification system which went live in early 2020 and is currently working with seven airlines at Robert Mugabe International Airport in Harare. The government also has plans to introduce a paper document carrying a quick-response (QR) code and, in April, announced that it will be issuing a COVID-19 pass in the form of an electronic card bearing a QR code that can be scanned for verification.

While such initiatives may make it harder for fake pass and fake test vendors to ply their trade at the Roadport terminal, it probably won’t deter them altogether. As Mchechesi himself acknowledges, QR codes can be falsified.

Meanwhile, the motivation to buy fake cards and test certificates will still be there. Says Mchechesi: “One of the things that struck me when we went to the terminal was that many of the people carrying fake cards did not know why they needed a vaccination. What they did know was that they needed the card if they wanted to do business or see loved ones on the other side of the border. They needed the card to get on the bus.”

**Figure:**
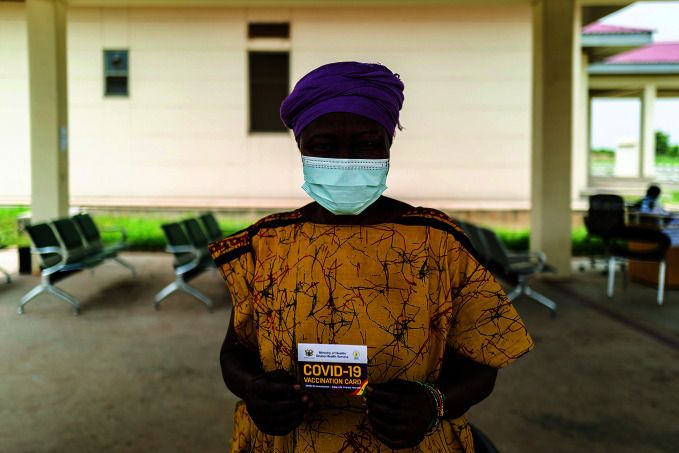
A woman holds up her vaccination card after receiving a dose of the AstraZeneca/Oxford COVID-19 vaccine at Accra's Ridge Hospital in Ghana.

WHO/Blink Media – Nana Kofi Acquah

**Figure:**
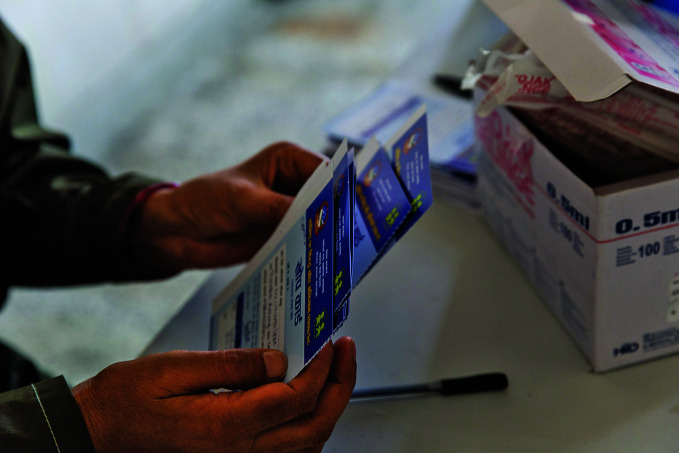
Health workers arrange vaccination cards at Paropakar Maternity and Women’s Hospital in Kathmandu, Nepal.

WHO/Blink Media – Uma Bista

